# Multi‐omics Insights Into the Effect of Feeding Yeast Culture on the Liver Metabolism and Immunity of *Plectropomus leopardus*

**DOI:** 10.1155/anu/6228089

**Published:** 2026-07-17

**Authors:** Chenlin Yin, Bo Wang, Haizhan Tang, Tongyao Zhang, Zhongyi Zhai, Jiahang Li, Chaofan Jin, Zhenmin Bao, Jingjie Hu

**Affiliations:** ^1^ MOE Key Laboratory of Marine Genetics and Breeding, College of Marine Life Sciences/Key Laboratory of Tropical Aquatic Germplasm of Hainan Province, Sanya Oceanographic Institution, Ocean University of China, Qingdao/Sanya, China, ouc.edu.cn; ^2^ Southern Marine Science and Engineer Guangdong Laboratory, Guangzhou 511458, China

**Keywords:** hepatic physiology, immune response, multiomics, *Plectropomus leopardus*, yeast culture

## Abstract

Yeast culture (YC) is widely applied as a functional feed additive, yet the mechanisms by which it regulates hepatic health, metabolism, and immune capacity in teleosts remain unclear. Here, juvenile *Plectropomus leopardus* were fed diets supplemented with 2.0% (YC2.0) or 8.0% YC (YC8.0) for 60 days, followed by hepatic histophysiological assessment, liver transcriptomic/metabolomic profiling, and an in vitro hepatocyte challenge with nervous necrosis virus (NNV). YC2.0 pincreased lipid droplet accumulation, improved antioxidant status, and lower aspartate aminotransferase(AST)/alanine aminotransferase (ALT)/alkaline phosphatase (ALP) and MDA in the liver than the control (Con) and YC8.0 groups. RNA‐seq identified 915 hepatic differentially expressed genes (DEGs): YC2.0‐specific genes were enriched in steroid hormone biosynthesis, bile secretion, and rhythmic regulation, whereas YC8.0‐upregulated clusters were enriched in NOD‐like receptor and JAK–STAT signaling, hematopoietic cell lineage, and apoptosis, with elevated immune‐ and apoptosis‐related markers (*nlrp1*, *mcub*, *myc*, *map1lc3b*, and *gadd45β/γ*). Metabolomics identified 680 differential metabolites across the three groups, showing that YC2.0 predominantly enhanced purine/nucleotide and glycerophospholipid metabolism, while YC8.0 shifted toward amino acid‐centered pathways (arginine biosynthesis, histidine metabolism, and FoxO signaling). Multiomics integration revealed positive correlations of L‐aspartic acid (L‐Asp) and glutamic acid (Glu) with inflammatory DEGs, and of glycerophosphoethanolamine (Geptn) with apoptosis‐related DEGs. In vitro, YC pretreatment alleviated NNV‐induced cytopathic effects (CPEs) in hepatocytes, suppressed viral mRNA accumulation, and reduced the mRNA levels of inflammatory cytokines (*il1*β, *ifng*, *il6*, *il12a*, *il16*, and *tnfsf14*) and immune pathway related DEGs. Collectively, these results characterize dose‐dependent effects of YC on hepatic physiology and immune regulation, supporting its application as a functional aquafeed supplement.

## 1. Introduction

With societal development, increasing attention has been directed toward healthy dietary practices. As an essential component of the global blue food system, the demand for fish products continues to rise worldwide, and aquaculture has become one of the fastest‐growing sectors in food production [1, 2]. To meet the escalating global demand, intensive and high‐density farming systems have been widely adopted. However, the intensification of aquaculture has also introduced substantial challenges, including heightened stress and increased susceptibility to infectious diseases in farmed fish, which can compromise their health and growth and ultimately reduce overall production efficiency. To mitigate these adverse effects, the application of feed additives has emerged as a key strategy in modern aquaculture [3]. Currently, aquafeed additives are generally categorized into three groups: nutritional supplements (including vitamins, minerals, and amino acids); microbiological regulators (including probiotic strains such as *Bacillus* and *Lactobacillus*); and plant‐derived bioactive compounds (including polysaccharides, flavonoids, and other plant metabolites) [4]. The judicious use of these additives offers considerable benefits, including the potential to promote growth, alleviate oxidative stress, and enhance immune function.

Yeast culture (YC) is a probiotic‐associated feed additive produced through yeast fermentation and characterized as a complex natural fermentation product. YC is enriched in diverse bioactive constituents, including mannan oligosaccharides (MOSs), β‐glucans, bioactive peptides, and vitamins [5]. As a functional dietary supplement, YC has been reported to exert multiple physiological regulatory effects in aquatic animals. For instance, supplementation with optimal levels of YC has been reported to improve growth performance catfish (*Clarias gariepinus*) and shrimp (*Litopenaeus vannamei*) [6, 7]. Dietary YC could improve hepatic function in juvenile largemouth bass (*Micropterus salmoides*) fed high‐starch diets [8]. Moreover, YC has emerged as a promising functional ingredient with immunomodulatory properties and the potential to improve fish health status. For example, YC has been shown to enhance immune responses in Atlantic salmon (*Salmo salar*), rainbow trout (*Oncorhynchus mykiss*), and shrimp [9–11], thereby improving their resistance to diverse bacterial and viral pathogens. These findings have provided valuable insights into the application of YC in aquaculture. However, our understanding of how YC influences the physiological status of aquatic organisms remains limited, as most studies have focused to phenotypic observations and enzyme activity measurements [12, 13]. With the advancement of high‐throughput sequencing technologies, multiomics approaches such as transcriptomics and metabolomics have been widely employed to identify key genes and regulatory networks involved in immunity, reproduction, and environmental stress responses in aquaculture species [14–16]. However, the application of multiomics approaches to elucidate the effects of YC on the cultured fish remains scarce.

The leopard coral grouper (*Plectropomus leopardus*), a highly valued coral reef species, is widely distributed across tropical and subtropical waters [17]. This species is characterized by its vivid body coloration, delicate flesh, and high protein content, which together confer substantial commercial value [18]. In recent years, the aquaculture of *P. leopardus* has progressed steadily, establishing it as one of the most economically important marine fish species in tropical aquaculture [19, 20]. However, intensive and high‐density aquaculture practices have resulted in the continuous exposure of *P. leopardus* to stressful conditions, leading to physiological alterations closely associated with oxidative stress, inflammatory responses, and metabolic dysregulation [14, 21–23]. In terms of feed additives, astaxanthin and Antarctic krill have been demonstrated to be suitable diet supplements for *P. leopardus* physiological health and body coloration [14, 24, 25]. YC, has emerged as promising functional additive in aquaculture; however, systematic investigations into its effects on this species are still limited. In this study, the effects of dietary YC with different levels (2%, named YC2.0, and 8%, named YC8.0) liver functions of *P. leopardus* was systematically investigated. The histological and enzyme analysis demonstrated the effects of dietary YC on lipid metabolism, antioxidant capacity, and immunity in the liver of *P. leopardus*. Integrated transcriptomic and metabolomic analyses revealed clear dose‐dependent responses: YC2.0 primarily enhanced metabolic and steroid biosynthetic pathways, whereas YC8.0 preferentially activated apoptosis‐ and immune‐related signaling. Notably, L‐aspartic acid (L‐Asp) and glutamic acid (Glu) were significantly correlated with differentially expressed genes (DEGs) associated with inflammation and apoptosis. Moreover, YC treatment attenuated nervous necrosis virus (NNV) infection in vitro in hepatocytes and modulated the expression of inflammatory cytokines as well as representative genes within transcriptomics‐identified pathways. These findings advance mechanistic understanding of how YC regulates liver health and immunity in *P. leopardus* and provide a scientific basis for its rational application in aquaculture.

## 2. Materials and Methods

### 2.1. Ethical Statement

This study was approved by the Institutional Animal Care and Use Committee of the College of Marine Life Sciences, the Ocean University of China on October 10, 2018 (Approval Number 20181010).

### 2.2. Diet Preparation

YC (derived from *Saccharomyces cerevisiae*) was supplied by the Beijing Enhalor Biotechnology Co., Ltd. (Beijing, China). Three experimental diets were prepared with graded YC supplementation levels of 0, 20 and 80 g/kg, designated as the control (Con), YC2.0, and YC8 groups, respectively. The nutrient composition of each diet was summarized in Supporting Information 1: Table S1. Diet manufacturing followed established procedures described previously [26]. After pelleting, the diets were air‐dried at ambient temperature, sealed in plastic bags, and stored at 20°C until feeding.

### 2.3. Feeding Experiment

A total of 270 healthy *P. leopardus* with uniform body weight (27.87 ± 0.73 g) obtained from the Hainan Chenhai Aquatic Products Co., Ltd. (Hainan, China) were randomly divided into three groups, each with three replicates (30 fish per tank), and reared in nine 400 L aquaria. Each tank was equipped with an independent seawater recirculating filtration system and an aeration device. Throughout the experiment, water temperature was maintained at 25–28°C, salinity at 30%–33%, pH at 7.7–8.0, and dissolved oxygen at 6.0–7.0 mg/L under a natural photoperiod. Fish were fed to apparent satiation three times daily (08:00, 12:00, and 17:00, Beijing time). Feeding was stopped once uneaten feed was observed in the outlet tank. Thirty minutes after each feeding, residual feed and feces were removed via the drainage system to maintain optimal water quality. The feeding trial lasted for 60 days.

### 2.4. Sample Collection

At 60 day postfeeding trial, all fish were anesthetized with MS‐222 (250 mg/L). Subsequently, the liver of nine individuals from each group were sampled. One portion of sample was immediately frozen in liquid nitrogen and stored at −80°C. The other portion was fixed in 4% paraformaldehyde (PFA) for 24 h, progressively dehydrated through an ethanol series (30%, 50%, 70%, 80%, 90%, and 100%), and preserved in absolute ethanol for histological observation.

### 2.5. Histological Observation

The liver tissue was dehydrated in ethanol, cleared in xylene, and embedded in paraffin. The tissue blocks were then cut into 5 μm thickness and stained with hematoxylin and eosin (H&E) using a commercial staining kit (C0105M, Beyotime Biotechnology, Shanghai, China). Histological images were acquired with a high‐throughput digital pathology system (Aperio GT450, Leica, Germany).

### 2.6. Enzyme Activity Measurement

Hepatic Na^+^/K^+^‐ATPase (NKA), fatty acid synthase (FAS), and glucose‐6‐phosphate dehydrogenase (G6PDH) activities were measured using commercial assay kits (Catalog number: BC0065, BC0550, and BC0260; Solarbio, Beijing, China, respectively). Total superoxide dismutase activity (T‐SOD), catalase (CAT), total glutathione peroxidase (T‐GPx), and total antioxidant capacity (T‐AOC) were determined using assay kits (Catalog number: S0101S, S0051, S0059S, and S0121, respectively; Beyotime Biotechnology, Shanghai, China). Aspartate aminotransferase (AST) activity, alanine aminotransferase (ALT) activity, alkaline phosphatase (ALP) activity, and malondialdehyde content were measured by the commercial enzyme assay kits (Catalog number: P2715, P2711, P0321, and S0131, respectively) from Beyotime Biotechnology (Shanghai, China). All assays were performed strictly according to the manufacturers’ instructions.

### 2.7. RNA Extraction, Transcriptomic Sequencing, and Analysis

The liver samples of nine individuals from each group (Con, YC2.0, and YC8.0 groups) was individually pooled in equal weight, with every set of three individuals, respectively. Total RNA was extracted using TRIzol reagent (Invitrogen, Carlsbad, CA, USA). Genomic DNA was eliminated through treatment with DNase I (TaKaRa, China). RNA quality and quantity were verified by 1.5% agarose gel electrophoresis and Agilent 2100 Bioanalyzer (Agilent Technologies, Santa Clara, CA, USA). The RNA with RIN value > 7.0 was used for cDNA library construction. Sequencing was performed on the Illumina NovaSeq 6000 platform, generating 150 bp paired‐end reads.

The quality of raw data were initially assessed using FastQC. After quality trimming with Trimmomatic v0.36, clean reads were aligned to the *P. leopardus* genome coding sequences [17] using salmon for transcript quantification, yielding raw counts and transcripts per million (TPM) values. Differential expression analysis was conducted using DESeq2 (|log2FoldChange| ≥ 1, *p* < 0.05). Time‐series expression patterns of DEGs were analyzed using the Mfuzz package in R software. KEGG pathway and GO term enrichment analyses were performed and visualized through the Omicshare platform (https://www.omicshare.com/tools/).

### 2.8. Quantitative Real‐Time PCR (qRT‐PCR)

The expression profiles of selected DEGs in liver tissues were validated by qRT‐PCR. Gene‐specific primers were designed using NCBI Primer‐BLAST (https://www.ncbi.nlm.nih.gov/) and are listed in Supporting Information 2: Table S2. The 10 μL reaction system contained SYBR qPCR SuperMix Plus (Sangon Biotech, China), 0.4 μL of each primer (10 μM), 2 μL cDNA template (10 ng), and 7.2 μL nuclease‐free water. Amplification was performed with initial denaturation at 95°C for 3 min, followed by 45 cycles of 95°C for 5 s and 60°C for 20 s. The *b2m* gene was used as the internal reference for normalization [27]. Each experiment was performed in triplicates.

### 2.9. In Situ Hybridization (ISH) and TUNEL Assay

Digoxigenin‐labeled RNA probes targeting the genes analyzed in this study were generated using a DIG RNA Labeling Kit (Roche). ISH was performed according to established laboratory protocols described previously [14]. Signal detection was carried out using a Nikon Eclipse Ti‐U microscope equipped with a 40× objective, and blue–purple staining was interpreted as a positive hybridization signal.

### 2.10. Liver Metabolites Profiling

Liver tissues (50 mg) from six individuals per group Con, YC2.0, and YC8.0 groups) were homogenized in 400 μL extraction buffer, incubated at −20°C for 30 min, and centrifuged prior to LC–MS analysis. Raw data were processed by removing missing values (80% rule) with QC validation (CV ≤ 30%). Multivariate statistical analysis included partial least squares‐discriminant analysis (PLS‐DA) with a 95% confidence level to assess metabolic differences among groups. Metabolites were considered significantly altered based on a variable importance in projection (VIP) score >1 and *p* < 0.05. Metabolic pathway annotation was performed using KEGG database (https://www.kegg.jp/), with all analyses conducted on the Majorbio Cloud Platform (https://cloud.majorbio.com).

### 2.11. In Vitro Experiment

Liver cells of *P. leopardus* were derived from an established passaged cell line of our lab. NNV was isolated from *P. leopardus* juveniles infected with NNV. Liver cells were seeded into 6‐well culture plates and cultured until 70%–80% confluence. The culture medium was then replaced with fresh medium containing YC at final concentrations of 0.05, 0.1, or 0.2 g/L, while an equal volume of PBS was added to the Con group. After 24 h of treatment, cells were infected with NNV at a multiplicity of infection (MOI) of 1. Following 48 h of incubation, total RNA was extracted from the cells for subsequent analysis.

### 2.12. Statistical Analysis

All the data were analyzed by one‐way ANOVA using IBM SPSS Statistics 26.0 (SPSS Inc., Chicago, IL, USA). Differences were considered statistically significant at *p* < 0.05. Results are expressed as mean ± standard error of the mean (SEM).

## 3. Results

### 3.1. Liver Histology and Physiological Status of *P. leopardus*

**Figure 1:**
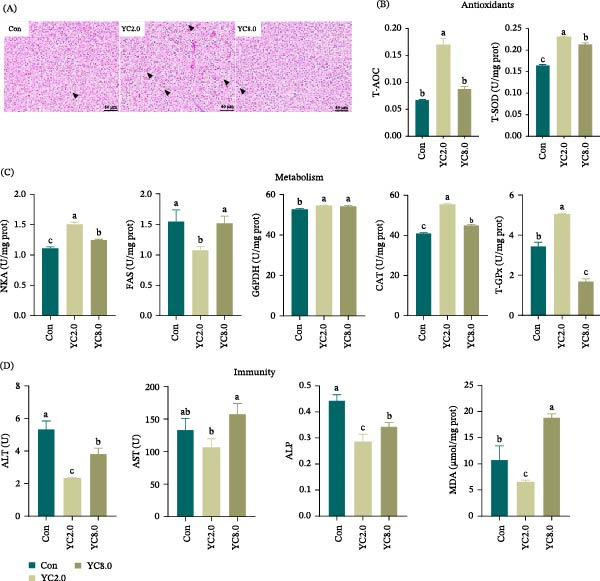
The liver histology and physiological status of *P. leopardus*. (A) The HE results of livers from different groups. Red arrows indicate lipid droplets. Scale bar = 60 μm. (B) Enzyme activities for antioxidants measured in liver tissues of individuals (*n* = 6) from each treatment group. (C) Enzyme activities for metabolism measured in liver tissues of individuals (*n* = 6) from each treatment group. (D) Enzyme activities for immunity measured in liver tissues of individuals (*n* = 6) from each treatment group. Different letters indicate the significant difference between groups.

H&E staining results exhibited that lipid droplet accumulation was markedly increased in the YC2.0 group compared to the Con group, whereas hepatic lipid content was notably reduced in the YC8.0 group (Figure 1A). Moreover, the contents associated with antioxidants, metabolism, and immunity in livers were analyzed. For antioxidants, the T‐AOC, T‐SOD, and CAT were all significantly increased (*p* < 0.05) in both YC2.0 and YC8.0 groups compared to Con group, with the YC2.0 group exhibiting the greatest overall enhancement. The activity of T‐GPx was upregulated in the YC2.0 group while downregulated in the YC8.0 group (Figure 1B). For metabolism, dietary 2.0% YC significantly enhanced hepatic NKA activity, while inhibited FAS activity compared with the Con and YC8.0 groups. Moreover, G6PDH activity was significantly upregulated in both YC2.0 and YC8.0 groups (Figure 1B). In terms of immunity, the activities of AST, ALT, ALP, and MDA content in the YC2.0 group were significantly lower than those in the Con and YC8.0 groups (*p* < 0.05). In contrast, AST activity and MDA levels were highest in the YC8.0 group (Figure 1D).

### 3.2. Identification of DEGs in *P. leopardus* Liver That Response to Dietary YC With Different Concentrations

**Figure 2:**
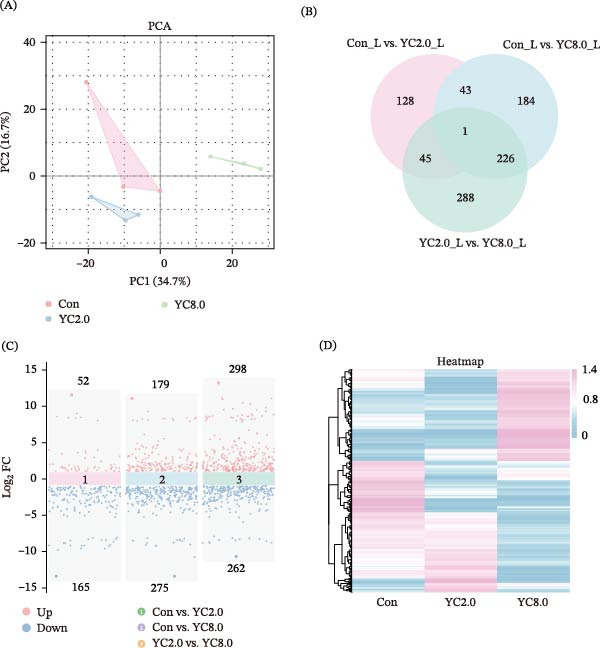
DEGs analysis of the livers in *P. leopardus* fed different concentrations of YC for 60 days. (A) Principal component analysis (PCA) of the liver samples from different groups. (B) Venn diagram for commonly and exclusively DEGs in three comparison groups. (C) Number of DEGs in three comparison groups. (D) Heatmap of DEGs among these three groups.

Subsequently, transcriptomic sequencing was conducted on liver samples from the Con, YC2.0, and YC8.0 groups. Principal component analysis (PCA) demonstrated distinct clustering of liver samples among the three treatment groups (Figure 2A), indicating clear transcriptomic differentiation induced by dietary YC supplementation. DEGs analysis across the three pairwise comparisons (Con vs. YC2.0, Con vs. YC8.0, and YC2.0 vs. YC8.0) identified a total of 915 DEGs in the liver, among which, only 1 DEG was shared in the three comparison groups (Figure 2B). The numbers of up‐ and downregulated DEGs for each comparison are illustrated in volcano plots (Figure 2C). The expression patterns of the DEGs among the three groups were further visualized using a heatmap (Figure 2D).

### 3.3. The Molecular Basis in the Liver That Response for Dietary YC at Different Concentrations

**Figure 3:**
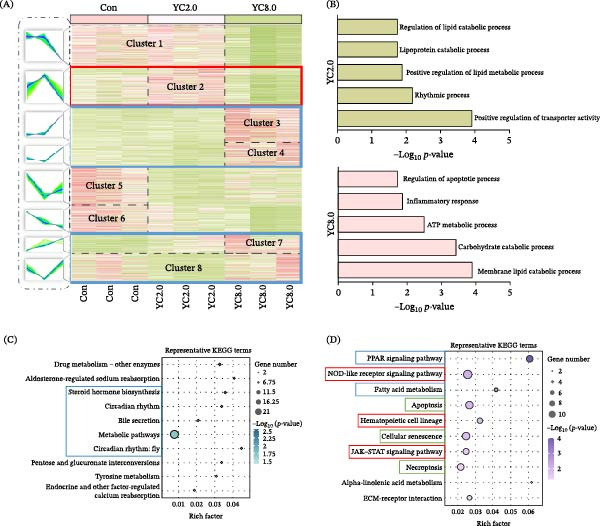
Functional enrichment analysis and expression patterns of DEGs in *P. leopardus* liver fed with different YC concentrations for 60 days. (A) Trend analysis of all DEGs based on their expression patterns. (B) GO functional enrichment analysis of DEGs. (C) KEGG pathway enrichment analysis of DEGs from cluster 2. (D) KEGG pathway enrichment analysis of upregulated DEGs in the YC8.0 group.

Trend analysis of all 915 liver DEGs identified eight distinct expression clusters (Figure 3A and Supporting Information 3: Figure S1). Among these, genes in cluster 2 were specifically upregulated in the YC2.0 group, whereas clusters 3, 4, 7, and 8 exhibited markedly elevated expression in the YC8.0 group. GO enrichment analysis indicated that cluster 2 DEGs were significantly enriched in terms related to transport regulation, rhythm, and lipid catabolism (Figure 3B). For clusters 3, 4, and 7, the DEGs were significantly associated with apoptosis, inflammatory process, and lipid catabolism (Figure 3B). Similarly, KEGG pathway analysis showed that DEGs from cluster 2 were primarily involved in steroid hormone biosynthesis, bile secretion, and general metabolic pathways (Figure 3C). However, DEGs from clusters 3, 4, and 7 were significantly enriched in pathways related to fatty acid metabolism and immune regulation, including the NOD‐like receptor signaling pathway, hematopoietic cell lineage, JAK–STAT signaling pathway, and apoptosis (Figure 3D).

Subsequently, the expression levels of representative DEGs involved in key pathways (Figure 4A) were validated. The expressions of these DEGs derived from qRT‐PCR results were highly consistent with the RNA‐seq data (Figure 4B, D). For instance, DEGs involved in steroid hormone synthesis and bile acid secretion (*ugt*, *slc22a2*, *cyp7a1*, and *atp1b4*) were significantly upregulated in the YC2.0 group. However, the DEGs from PPAR signaling pathway and fatty acid metabolism (*cpt1*, *fabp1*, *fasn*, and *cyp4b1*) exhibited highest expression values in YC8.0 group. ISH results indicated the positive signals of *cyp7a1* were strongest in YC2.0 group, while the signals of *fasn* and *cpt1* were most obvious in YC8.0 group (Figure 4C). For DEGs involved in immune related pathways, including *merk*, *pim3*, *cdkn1d*, *myc*, *map1lc3b*, *mcub*, *nlrp1*, *itgαv*, *facl5*, and *cd36*, were all markedly upregulated in the YC8.0 group. Notably, pro‐apoptotic genes (*gadd45*β and *gadd45*γ) also showed significantly increased expression in the YC8.0 group (Figure 4D). Furthermore, ISH results revealed that positive signals of *gadd45*γ were markedly stronger in the liver of the YC8.0 group (Figure 4E). Consistently, the TUNEL assay demonstrated the highest levels of apoptosis in the liver of the YC8.0 group (Figure 4E).

Figure 4Validation of the representative DEGs expressions and TUNEL assays. (A) The expressions of representative DEGs from key pathways. (B) qRT‐PCR validation of expressions of DEGs associated with metabolism. Data were shown by mean ± SEM (*n* = 3). (C) ISH validation of the expressions of *cyp7a1*, *fasn*, and *cpt1*. I, cell nucleus; II, red blood cells; III, stellate cells; and the green circles indicate hepatic sinusoids. The positive signals were indicated by arrows. Scale bar: 20 μm. (D) qRT‐PCR validation of expressions of DEGs associated with immunity and apoptosis. Data were shown by mean ± SEM (*n* = 3). (E) ISH validation of the expressions *gadd45*γ and TUNEL analysis. I, cell nucleus; II, red blood cells. Scale bar: 20 μm. The positive signals were indicated by arrows.

**Figure:**
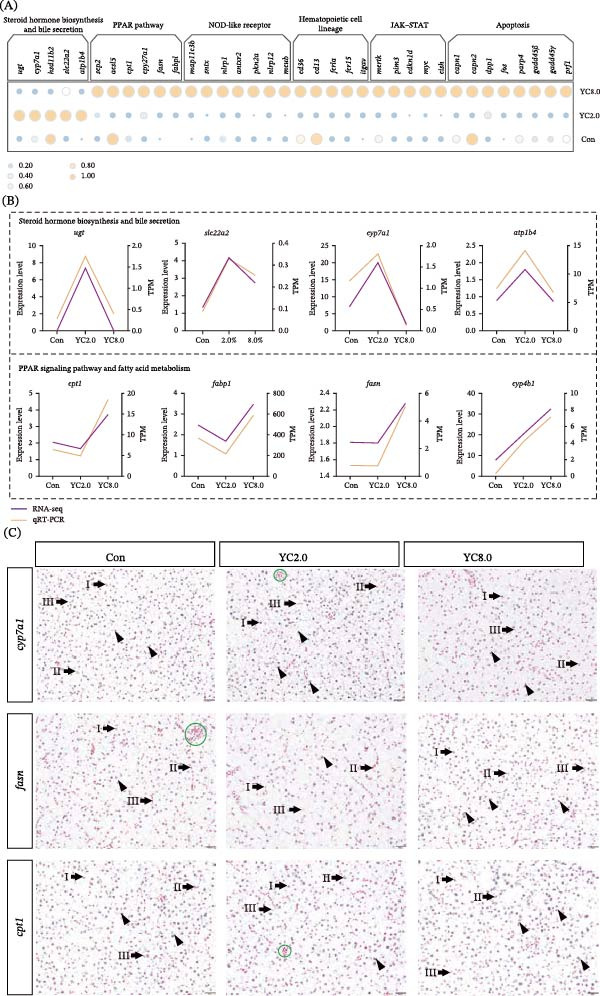

**Figure:**
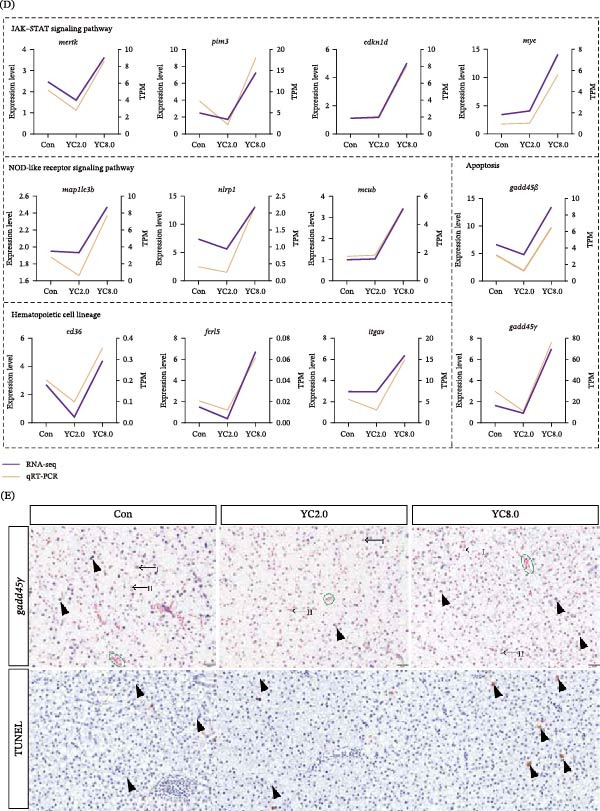

### 3.4. Differential Metabolite Identification in the Liver

**Figure 5:**
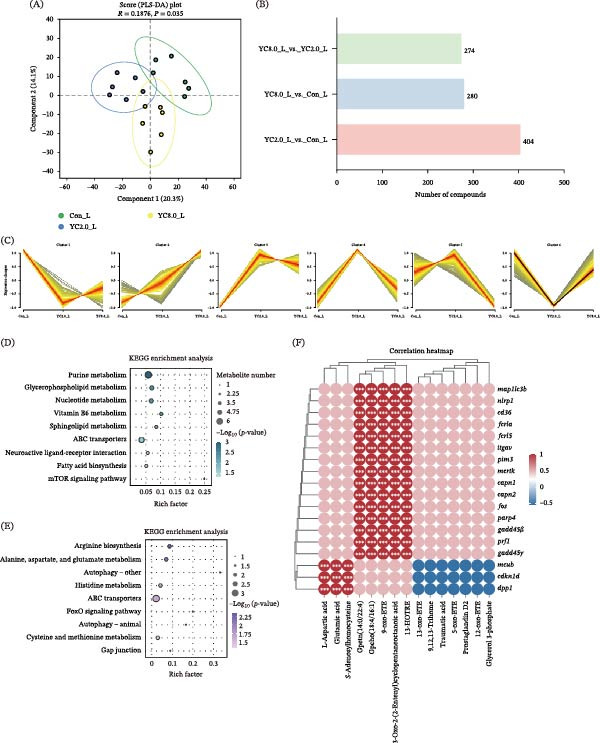
Hepatic metabolite enrichment patterns and functional analysis of DMs in *P. leopardus* fed YC with different concentrations. (A) Partial least squares‐discriminant analysis (PLS‐DA). (B) The number of DMs between each comparison group. (C) Mfuzz clustering analysis of DMs. (D) KEGG pathway enrichment of upregulated DMs (YC2.0 group). (E) KEGG pathway enrichment of upregulated DMs (YC8.0 group). (F) Spearman’s correlation analysis between DEGs and DMs involved in hepatic bile secretion and lipid metabolism. Correlation coefficients were color‐coded (red: positive; blue: negative).  ^∗^ *p* < 0.05,  ^∗∗^ *p* < 0.01, and  ^∗∗∗^ *p* < 0.001.

PLS‐DA was conducted on hepatic metabolites of *P. leopardus* following feeding with different YC concentrations. The PLS‐DA plot revealed significant intergroup differences (*R* ^2^ *X*(cum) = 0.733; *R* ^2^ *Y*(cum) = 0.992; *Q* ^2^(cum) = 0.817) (Figure 5A). Differential metabolite analysis among three comparison groups yiled a total of 680 DMs across the Con, YC2.0, and YC8.0 groups (Supporting Information 3: Figure S1A). The up‐ and downregulated metabolites were concluded in Figure 5B. Cluster analysis of the top 200 metabolites showed distinct grouping within each sample group, indicating excellent experimental reproducibility (Supporting Information 3: Figure S1B).

### 3.5. Functional Analysis of DMs and Their Correlation With DEGs

Trend analysis of all DMs generated six distinct clusters (Figure 5C). KEGG compound classification revealed that the upregulated metabolites were predominantly enriched in lipid, nucleic acid, and organic acid metabolic pathways (Supporting Information 4: Figure S2A). The metabolites upregulated in YC8.0 group were primarily associated with neurotransmitters, lipids, and nucleic acids (Supporting Information 4: Figure S2B). KEGG pathway enrichment results showed that upregulated DMs in YC2.0 group were significantly involved in lipid metabolic pathways, including purine metabolism, nucleotide metabolism, glycerophospholipid metabolism, lipid synthesis, and vitamin B6 metabolism (Figure 5D). In contrast, upregulated metabolites in YC8.0 group were predominantly associated with amino acid metabolic pathways, including arginine biosynthesis, histidine metabolism, FoxO signaling, mTOR signaling, and ABC transporters (Figure 5E). Furthermore, correlation analysis revealed that L‐Asp and Glu were significantly positively correlated with multiple inflammatory DEGs. The glycerophosphoethanolamine (Geptn) showed a strong positive correlation with several apoptosis‐related genes, including *map1lc3b*, *gadd45*β, and *gadd45*γ. In addition, genes positively correlated with L‐Asp and Glu expression were predominantly enriched in inflammation‐associated pathways, such as the NOD‐like receptor signaling pathway and the JAK–STAT signaling pathway (Figure 5F).

### 3.6. YC Attenuates NNV Infection in Hepatocytes via Immune Regulation In Vitro

**Figure 6:**
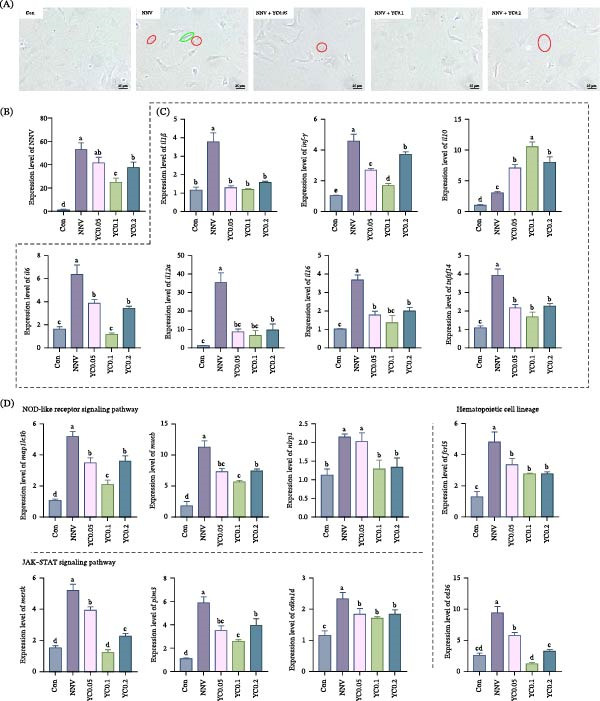
The effects of YC on NNV infection in hepatocytes via immune regulation in vitro. (A) The morphology of hepatocytes from different groups. Scale bar: 20 μm. (B) The mRNA level of NNV in the hepatocytes from different groups. (C) The expression levels of inflammatory factors in the hepatocytes from different groups. (D) The expression levels of representative DEGs derived from transcriptome data in the hepatocytes from different groups. Data were shown by mean ± SEM (*n* = 3). Different letters indicate the significant difference between groups.

The above results demonstrated that dietary YC exerts regulatory effects on hepatic metabolism and immunity. To further elucidate the role of YC in hepatic immune regulation, its effects on NNV infection in hepatocytes were subsequently examined in vitro. Compared to the Con group, hepatocytes exposed to NNV exhibited pronounced cytopathic effects (CPEs) at 48 h post‐infection, including extensive cytoplasmic vacuolation accompanied by partial cell lysis and detachment. In YC treated group, these pathological features were markedly alleviated, with the most pronounced protective effect observed in the YC0.1 group (Figure 6A). Meanwhile, the mRNA levels of NNV in hepatocytes from YC treated group were significantly downregulated compared to NNV group (Figure 6B). Furthermore, the expressions of some representative inflammatory factors, such as *il1*β, *infγ*, *il10*, *il6*, *il12a*, *il16*, and *tnfsf14*, were found to be significantly downregulated compared to the NNV group (Figure 6C). To further investigate the regulatory roles of YC on immune response induced by NNV, the expressions of representative DEGs involved in immune related pathways identified in the transcriptome were examined. The results indicated that these DEGs were all significantly upregulated in NNV group compared to the Con group. However, the expression levels of these DEGs were downregulated in YC treated group compared to NNV group (Figure 6D).

## 4. Discussion

In this study, the effects of dietary YC on hepatic physiology, metabolism, and immune regulation in *P. leopardus* were systematically investigated. As a central metabolic and immune organ, the fish liver sensitively reflects dietary adaptation through its structure and physiology [14, 28–30]. *P. leopardus* from YC2.0 group exhibited pronounced hepatic lipid accumulation, whereas hepatic lipid content was markedly lower in YC8.0 group. Although studies in mammals often report YC‐mediated reductions in hepatic lipid deposition [31, 32], the divergent outcomes here likely reflect species‐specific physiology and differential energy allocation across supplementation levels under nutrient‐replete conditions. Previous studies have shown that appropriate YC supplementation can enhance antioxidant capacity and thereby improve the physiological status of aquatic animals [5, 33]. In the present study, fish fed with the 2.0% YC diet exhibited significantly higher hepatic T‐AOC, T‐SOD, and CAT activities, suggesting robust activation of the antioxidant defense system, which is consistent with results reported in *P. ussuriensis* [33] demonstrating that the mannans and *β*‐glucans abundant in YC might play a key role in antioxidant system [33]. In contrast, YC8.0 might have promoted excessive ROS generation, overwhelming antioxidant capacity, depleting enzymes such as SOD, and triggering OS, ultimately impairing health. Moreover, dietary YC has also been identified to be beneficial for nonspecific immunity. For instance, the ALT, AST, and MDA contents in *Epinephelus fuscoguttatus♀* × *Epinephelus lanceolatus♂* were significantly downregulated with dietary 4% YC. In this study, the contents of ALT, AST, ALP, and MDA were lowest in YC2.0 group, suggesting the YC could be ideal feed additives for enhancing nonspecific immunity. However, the optimal dose might divergent among different groups.

In this study, comparative transcriptomic analysis was performed on the livers of *P. leopardus* from different dietary groups. In the YC2.0 group, the upregulated DEGs were significantly enriched in metabolism‐related pathways. Among these, steroid hormone biosynthesis, bile secretion, and circadian rhythm pathways were indirectly associated with immune regulation. Previous studies have demonstrated that *ugt* and *cyp7a1*, key genes in the steroid hormone biosynthesis pathway, play important roles in anti‐inflammatory responses [34, 35]. Moreover, appropriate bile acid synthesis (*ugt*, *cyp7a1*) can improve hepatic inflammation [35]. Feed formulations are also known to exert synergistic effects on the circadian system and nutrient metabolism in fish, and the bidirectional relationship between circadian rhythms and inflammatory responses has been documented [36, 37]. Notably, DEGs such as *per2* and *nr1d2* in the circadian rhythm pathway have been shown to alleviate hepatic inflammation and enhance metabolism [38, 39]. In contrast, the upregulated DEGs in the YC8.0 group were significantly enriched in inflammation‐ and cell process‐related pathways. The NOD‐like receptor signaling pathway [40] and JAK–STAT signaling pathway [41] are frequently activated during inflammation and immune responses, and several proinflammatory genes—including *map1lc3b*, *mcub*, *nlrp1*, *cdkn1d*, *pim3*, *myc*, and *mertk*—were upregulated. Importantly, *itgαv* and *cd36* were upregulated and enriched in the hematopoietic cell lineage pathway, which plays a crucial role in liver repair processes [11, 42].

Metabolic status is closely linked to physiological conditions, and metabolomic analysis has therefore been recognized as a reliable tool for identifying biomarkers associated with organ physiological status [43]. In the present study, metabolomic analysis revealed that YC supplementation significantly affected the hepatic metabolic profile of *P. leopardus*. In the YC2.0 group, the upregulated DMs were significantly enriched in several high‐efficiency metabolic and signal transduction pathways, including purine metabolism [44, 45], nucleotide metabolism [46], glycerophospholipid metabolism [47], lipid biosynthesis [48], and vitamin B6 metabolism [49]. The activation of these pathways suggests that the liver may have been in a state of accelerated synthesis of proteins, membranes, and nucleic acids, thereby facilitating rapid growth. In addition, enrichment in the mTOR signaling pathway [50] and ABC transporters [51] may indicate enhanced metabolic regulation and cytoprotective capacity in the liver. In the YC8.0 group, the upregulated DMs included L‐Asp and Glu, two key amino acids in nitrogen metabolism [52, 53], which were enriched in multiple stress‐ and inflammation‐related pathways such as arginine biosynthesis [54], histidine metabolism [55], and the FoxO signaling pathway [56]. Moreover, elevated levels of L‐Asp and S‐adenosylhomocysteine (SAH) in the cysteine and methionine metabolism pathway suggested that the liver might have been under intensified oxidative stress [57]. Collectively, these metabolic alterations indicated that the livers of *P. leopardus* in the YC8.0 group underwent metabolic reprograming driven by low‐grade inflammation or stress. Furthermore, Geptn, a metabolite derived from phospholipid degradation and involved in autophagy processes [58], was upregulated in YC8.0 group, suggesting a potential shift in cellular processes toward apoptosis.

Integrative analysis of transcriptomic and metabolomic data revealed significant coordinated alterations in inflammation and apoptosis‐related pathways, with DEGs and DMs. Notably, the nitrogen‐metabolism amino acids L‐Asp and Glu exhibited a significant positive correlation with inflammation‐associated genes such as *nlrp1*, *mcub*, *pim3*, *cdkn1d*, and *myc*, all of which are critical in mediating the NOD‐like receptor and JAK–STAT signaling pathways [40, 41, 59], suggesting that dysregulation of amino acid metabolism might be closely associated with inflammatory responses.

The in vitro hepatocyte infection model further explored the immunomodulatory effects of YC. NNV infection typically induces characteristic CPEs, including extensive cytoplasmic vacuolation, cell rounding, cell detachment, and eventual disruption of the cell monolayer, which are closely associated with endosome/lysosome‐derived vacuoles and virus‐induced cell death programs [1–3]. In the present study, YC pretreatment markedly alleviated these pathological changes and simultaneously reduced intracellular NNV mRNA levels, suggesting that YC may confer protection by restricting viral replication and/or enhancing cellular tolerance to infection [4–6]. As an important immune organ in fish, the liver enables hepatocytes to sense viral nucleic acids through pattern‐recognition receptors (PRRs) and thereby trigger downstream inflammatory responses; accordingly, the reduced expression of cytokines such as *il1*β, *il6*, and *infγ* after YC treatment may help prevent immunopathological damage caused by excessive inflammation [6–8]. This interpretation is consistent with previous studies showing that yeast‐derived functional components—particularly yeast cell wall β‐glucans, as well as MOS and nucleotides—can modulate innate immune signaling and cytokine profiles in fish, enhancing host defense while avoiding detrimental hyperinflammatory states [5, 6, 8]. Notably, β‐glucan has been demonstrated to enhance resistance of groupers to NNV, accompanied by reduced inflammasome associated caspase‐1/IL‐1β output and strengthened antiviral responses; moreover, yeast β‐glucan can enhance type I interferon–related antiviral programs and inhibit RNA virus replication in zebrafish in vivo and in fish cells in vitro [5, 6].

## 5. Conclusion

In summary, dietary YC exerted dose‐dependent effects hepatic physiology and immune responses in juvenile *P. leopardus*. YC2.0 increased hepatic lipid droplets, enhanced antioxidant capacity, and reduced AST/ALT/ALP and MDA relative to the Con and YC8.0. Transcriptomics identified 915 hepatic DEGs, with YC2.0 mainly enriching steroid hormone biosynthesis, bile secretion, and rhythmic regulation, whereas YC8.0 preferentially activated NOD‐like receptor and JAK–STAT signaling, hematopoietic cell lineage, and apoptosis. Metabolomics revealed 680 differential metabolites, showing that YC2.0 enhanced purine/nucleotide and glycerophospholipid metabolism, while YC8.0 shifted toward amino‐acid–centered pathways (arginine biosynthesis, histidine metabolism, and FoxO signaling). Multiomics integration linked L‐Asp and Glu to inflammatory DEGs and Geptn to apoptosis‐related DEGs. In vitro, YC pretreatment mitigated NNV‐induced CPEs, reduced viral mRNA, downregulated inflammatory cytokines and immune pathway related DEGs. Collectively, these results systematically characterize the impacts of YC on hepatic physiology and immune regulation, providing insights for its application as a functional aquafeed supplement.

## Author Contributions

**Chenlin Yin**: writing – original draft, visualization, software, methodology, investigation, formal analysis, conceptualization. **Bo Wang**: writing – review and editing, conceptualization, data curation, supervision, funding acquisition. **Haizhan Tang, Zhongyi Zhai, and Jiahang Li**: investigation, formal analysis. **Tongyao Zhang**: conceptualization, methodology. **Chaofan Jin**: writing – review and editing, data curation, supervision, methodology, funding acquisition. **Zhenmin Bao**: resources. **Jingjie Hu**: resources, project administration.

## Funding

This study was supported by the Key R&D Project of Hainan Province (Grant ZDYF2024XDNY275), the Natural Science Foundation of China (Grant 32503173), and the Project of Sanya Yazhou Bay Science and Technology City (Grant SKJC‐JYRC‐2025‐54). This work was supported by the High‐Performance Computing Platform of YZBSTCACC.

## Conflicts of Interest

The authors declare no conflicts of interest.

## Supporting Information

Additional supporting information can be found online in the Supporting Information section.

## Supporting information


**Supporting Information 1** Table S1: The diet composition used in this study.

**Supporting Information 2** Table S2: Primer information used in this study.

**Supporting Information 3** Figure S1: Hepatic metabolite enrichment patterns in *P. leopardus* fed YC with different concentrations. (A) Venn diagram of DMs among three comparison groups. (B) Hierarchical clustering heatmap of metabolites.

**Supporting Information 4** Figure S2: Functional analysis of DMs in *P. leopardus* fed YC with different concentrations. (A) KEGG compound classification of upregulated DMs (YC2.0 group). (B) KEGG compound classification of upregulated DMs (YC8.0 group).

## Data Availability

Data presented in this study will be available upon the request.
