# Supplementary material for: Multi‐omics Insights Into the Effect of Feeding Yeast Culture on the Liver Metabolism and Immunity of Plectropomus leopardus
Source: Aquac Nutr. 2026 Jul 17;2026:6228089. doi: 10.1155/anu/6228089 (PMC13377622; doi:10.1155/anu/6228089)
Supplement: Supplementary file 1 — Supporting Information 1 Table S1: The diet composition used in this study. [file ANU-2026-6228089-s001.docx]

**Table S1. Composition of the experimental diets**

| Item | group | | |
| --- | --- | --- | --- |
|  | Con | YC2.0 | YC8.0 |
| Fish meal | 46 | 46 | 46 |
| Shrimp meal | 3 | 3 | 3 |
| Soy protein concentrate | 16 | 14 | 8 |
| Yeast culture (YC) | 0 | 2 | 8 |
| Soy lecithin | 2 | 2 | 2 |
| Mineral premix^a^ | 1 | 1 | 1 |
| Vitamin premix^b^ | 1 | 1 | 1 |
| Wheat flour | 15 | 15 | 15 |
| Peanut meal | 11 | 11 | 11 |
| Fish oil | 3 | 3 | 3 |
| Choline chloride | 0.5 | 0.5 | 0.5 |
| Gelatin | 1.5 | 1.5 | 1.5 |

**a**

Mineral premix (mg kg⁻¹ diet): sodium fluoride (NaF), 4; potassium iodide (KI), 1.6; cobalt chloride hexahydrate (CoCl₂·6H₂O, 1%), 100; copper sulfate pentahydrate (CuSO₄·5H₂O), 20; ferrous sulfate monohydrate (FeSO₄·H₂O), 160; zinc sulfate monohydrate (ZnSO₄·H₂O), 100; manganese sulfate monohydrate (MnSO₄·H₂O), 120; magnesium sulfate heptahydrate (MgSO₄·7H₂O), 2400; calcium dihydrogen phosphate monohydrate [Ca(H₂PO₄)₂·H₂O], 6000; sodium chloride (NaCl), 200; zeolite powder, 30,900.

**b**

Vitamin premix (mg kg⁻¹ diet): thiamine (vitamin B₁), 25; riboflavin (vitamin B₂), 45; pyridoxine (vitamin B₆), 20; cyanocobalamin (vitamin B₁₂), 0.1; menadione (vitamin K₃), 10; myo-inositol, 800; pantothenic acid, 60; nicotinic acid, 200; folic acid, 1.2; biotin, 32; cholecalciferol (vitamin D₃), 5; DL-α-tocopheryl acetate (vitamin E), 120; ascorbic acid (vitamin C), 2000; choline chloride, 2000; ethoxyquin, 150; manna-croup, 14,520
