# Supplementary material for: Multi‐omics Insights Into the Effect of Feeding Yeast Culture on the Liver Metabolism and Immunity of Plectropomus leopardus
Source: Aquac Nutr. 2026 Jul 17;2026:6228089. doi: 10.1155/anu/6228089 (PMC13377622; doi:10.1155/anu/6228089)
Supplement: Supplementary file 2 — Supporting Information 2 Table S2: Primer information used in this study. [file ANU-2026-6228089-s002.docx]

Table S2**.** Primer information used in this study.

| Primer Name | Primer Sequence (5’-3’) |
| --- | --- |
| q_*cyp7a1*_f | TGCAAGATCGCAGGCCAATA |
| q_*cyp7a1*_r | GTGGCCAAATGCCTTGACAG |
| q_*ugt*_f | CGGCCTCTCATGCCTAACTTT |
| q_*ugt*_r | CACGTCTCCAAATCTTGCGG |
| q_ *map1lc3b*_f | GCTGTCAGAGGATCAGTGTCTC |
| q_ *map1lc3b*_r | TCATGTTGACGTGGTCTGGC |
| q_ *mcub*_f | GGGTTACCTCACCTGGTATGTG |
| q_ *mcub*_r | TGGTGAGGATGTAGTAGGCGA |
| q_ *nlrp1*_f | GACAAGCCCATGCTTTTGGG |
| q_ *nlrp1*_r | TCTGAGCTCTCGTGTGCTTG |
| q_ *cd361*_f | CATCTTTGAGCCGGCCATGT |
| q_ *cd361*_r | GAAGCGAATAAGCTCCAGCC |
| q_*gadd45β*_f | TGCATCCTTGTCACCAACCC |
| q_*gadd45β*_r | GCAGAAGCTGCTGATGCTCT |
| q_*gadd45γ*_f | TTGCATTCTTGTCACGAGCC |
| q_*gadd45γ*_r | CAGCGTTCTGGGAGTGTGAT |
| q_ *fcrl5*_f | TGAGTTTAACTGCCGCCGTG |
| q_ *fcrl5*_r | TTCAGACCACGCATCACTGG |
| q_ *itgav*_f | GCTCAACGTCTCGAACCCT |
| q_ *itgav*_r | CTCCGAATCTCGTTCCCTGG |
| q_ *pim3*_f | TCGCACATTTGCAACCCTTC |
| q_ *pim3*_r | CTTTTCCACTTTGACCGGCTG |
| q_ *cdkn1d*_f | CGTGATGCAAGCACAACAC |
| q_ *cdkn1d*_r | GCGCAACCAGAAGTAGGTCA |
| q_ *myc*_f | TGGATTCACCTGTTTCAGCCA |
| q_ *myc*_r | TTTCCTTATCGTGACGCTCG |
| q_ *mertk*_f | CTGAAGGCCTGCCCACATAA |
| q_ *mertk*_r | CGTTGGACTCCGTTCTCCTC |
| q_ *per2*_f | TTCCATTCGACAGGTCAGGC |
| q_ *per2*_r | GTTTCCGAGGCGTTTCGATG |
| q_ *nr1d2*_f | GTGGTCGACTTCGCCAAAAG |
| q_ *nr1d2*_r | CAAACGTTCCAGCCTTCAGC |
| q_ *b2m*_f | CCTTCAGAAACAACTGGCAATT |
| q_ *b2m* _r | GGCATATTCCTTAACTTTGGTCC |
