# Supplementary figures and images for: Multi‐omics Insights Into the Effect of Feeding Yeast Culture on the Liver Metabolism and Immunity of Plectropomus leopardus
Source: Aquac Nutr. 2026 Jul 17;2026:6228089. doi: 10.1155/anu/6228089 (PMC13377622; doi:10.1155/anu/6228089)

A

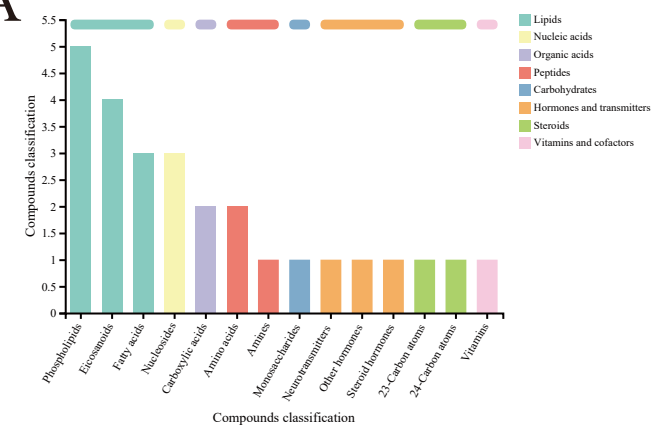

B

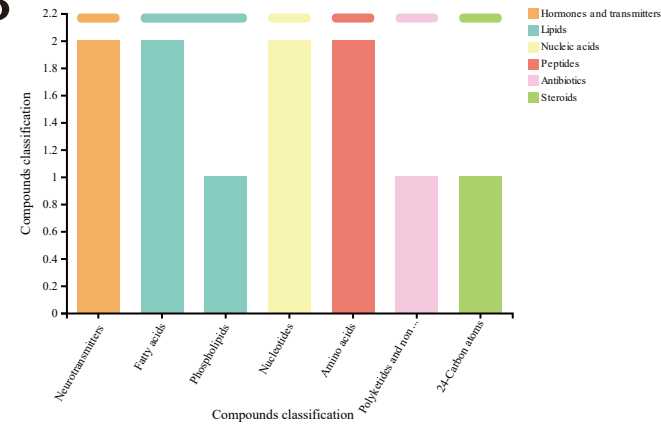

Supplement: Supplementary file 4 — Supporting Information 4 Figure S2: Functional analysis of DMs in P. leopardus fed YC with different concentrations. (A) KEGG compound classification of upregulated DMs (YC2.0 group). (B) KEGG compound classification of upregulated DMs (YC8.0 group). [file ANU-2026-6228089-s004.pdf]
