# Supplementary material for: Multi‐omics Insights Into the Effect of Feeding Yeast Culture on the Liver Metabolism and Immunity of Plectropomus leopardus
Source: Aquac Nutr. 2026 Jul 17;2026:6228089. doi: 10.1155/anu/6228089 (PMC13377622; doi:10.1155/anu/6228089)
Supplement: Supplementary file 3 — Supporting Information 3 Figure S1: Hepatic metabolite enrichment patterns in P. leopardus fed YC with different concentrations. (A) Venn diagram of DMs among three comparison groups. (B) Hierarchical clustering heatmap of metabolites. [file ANU-2026-6228089-s003.pdf]

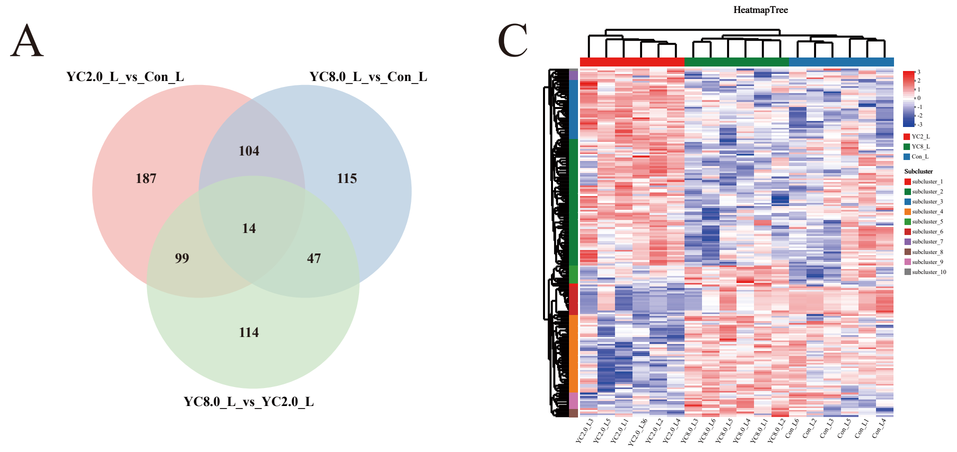

A
YC2.0_L_vs_Con_L
YC8.0_L_vs_Con_L
187
104
115
14
99
47
114
YC8.0_L_vs_YC2.0_L
C
HeatmapTree
3
2
1
0
-1
-2
-3
YC2_L
YC8_L
Con_L
Subcluster
subcluster_1
subcluster_2
subcluster_3
subcluster_4
subcluster_5
subcluster_6
subcluster_7
subcluster_8
subcluster_9
subcluster_10
